# Adherence to the Mediterranean Diet of Pregnant Women in Central South Africa: The NuEMI Study

**DOI:** 10.1177/11786388221107801

**Published:** 2022-06-24

**Authors:** Hermina Catharina Spies, Mariette Nel, Corinna May Walsh

**Affiliations:** 1 University of the Free State, South Africa

**Keywords:** Mediterranean Diet adherence, pregnant

## Abstract

**Introduction:**

The Mediterranean diet (MeD) has been shown to have significant health benefits for adults and children. A mother’s diet during pregnancy directly impacts the health of her offspring. This study aimed to investigate the adherence to the MeD of pregnant women attending antenatal care at a Regional Hospital in Bloemfontein, South Africa (SA).

**Methods:**

A cross-sectional study was conducted on a consecutive sample of 681 pregnant women who attended the antenatal clinic of a Regional Hospital in Bloemfontein. Socio-demographics included: age, highest level of education, household income, employment status, and income stability. Food group intake was assessed with a quantitative food frequency questionnaire. The adapted Mediterranean Diet Adherence Screener (MeDAS) consisted of 13 of the original 14 questions that measured intake of key food groups (score of ⩽7 poor, 8-9 moderate, ⩾10 good) (wine intake was excluded for pregnant women).

**Results:**

A total of 681 pregnant women with a median age of 31.8 years (IQR: 26.8-36.5 years) and a median gestational age of 32.0 weeks at the time of the interview participated in the study. The vast majority showed poor adherence to the MeD (99.6%), with only 0.4% (n = 3) having moderate adherence and 0% good adherence. The median adherence score was 5 points and the maximum 8 points. Of those with poor adherence, only 11.5% had tertiary education, 43.2% earned less than R 3000 (<201 USD) per month, 52.5% were unemployed, and 42.0% did not have a stable income in the past 6 months. Of the 3 participants with moderate adherence, all had grade 11 to 12 education, 2 out of the 3 earned more than R3000 (201 USD), one was unemployed, and 2 had a stable income over the past 6 months. Compared to those with an income ⩽ R3000 (⩽201 USD), those with an income above R3000 were significantly more likely to eat nuts (including peanuts) (2.0% vs 4.6%, *P* = .05), and adhere to sofrito (similar to tomato and onion relish) intake (9.2% vs 15.6%, *P* = .02). Compared to those who only had a primary education level up to grade 10 (n = 229), those who had a secondary education level or more (grade 11 and higher, n = 452) were significantly more likely to consume enough olive oil per day (1.3% vs 5.0%, *P* = .01), and to consume sofrito (6.6% vs 18.0%, *P* = .02).

**Conclusion:**

Pregnant participants showed poor adherence to the MeD. Although almost all women fell in the poor adherence group, secondary education contributed to consuming recommended amounts of olive oil and sofrito and higher income was associated with an adequate intake of nuts and sofrito. Based on the findings, we recommend the development of a contextualized MeDAS tool that includes foods that are typically eaten by most South Africans for similar MeD benefits.

## Introduction

A large body of evidence shows that the Mediterranean diet (MeD) is associated with multiple health benefits throughout all life stages,^1^ including pregnancy and fetus development. These benefits include: a lower risk of excess weight gain during pregnancy^2^; reduced incidence of gestational diabetes in mothers and congenital defects in offspring^3^; lower risk of small for gestational age (SGA) babies^4,5^; and protection against cardio-metabolic risk in the offspring.^6,7^

The MeD is characterized by increased consumption of unprocessed foods, plant foods such as fruit and vegetables, ﬁsh, nuts and legumes, and olive oil,^8^ making it rich in carbohydrates (specifically whole grains),^9^ ﬁber and antioxidants.^4^ In contrast, the intake of red meat, animal fats, sugars, and salt is lower.^8^ This diet is rich in mono-unsaturated fatty acids (MUFA) (present in olive oil, Brazil nuts, almonds, avocados, peanuts, and olives), omega-3 polyunsaturated fatty acids (omega-3 PUFA) (present in cold-water fatty fish, walnuts, canola oil, and flaxseed oil) and antioxidant polyphenols (present in berries, nuts, vegetables, legumes, red wine, onions, and garlic).^9^ The MeD also contains low-to-moderate amounts of dairy and eggs, and is low in saturated fatty acids (fat in red meat, cream, butter, cheese, and full cream dairy).^9^ In contrast to the MeD, the Western diet includes high intakes of refined carbohydrates, added sugars, fats (increased intake of take-aways/ fast foods), animal-source foods (red and processed meat), and salt.^10^ Consuming a Western diet has been positively associated with an increased risk for delivering SGA infants.^11^

To assess adherence to the MeD, a variety of scoring tools have been developed. These include the 9-item Mediterranean diet score (MDS) developed by Trichopoulou et al,^12^ the 14-food group Mediterranean Diet Serving Score (MEDSS) by Monteagudo et al,^13^ and the Mediterranean Diet Score for pregnant women (MDS-preg).^6^ The 14-item Mediterranean diet adherence screener (MeDAS)^14,15^ was first and habitually used in the “Prevención con Dieta Mediterránea” (PREDIMED) study in Spain from 2003 to 2011,^14^ which was designed to assess the long term effects of the MeD on the incidence of cardiovascular disease in men and women with a high cardiovascular risk.^16^

Although the anticipated benefits of a MeD are extensive, its implementation seems to be hindered by poor adherence.^17^ Several factors can influence the ability to adhere to the MeD guidelines. Cultural and socio-economic factors, including urbanization, have globally led people to transition from traditional healthier diets to an unhealthy Western-type diet.^10^ In addition, low education has also been linked to poor adherence to Mediterranean-like eating patterns.^18^

Moreover, the MeD may seem to be more expensive.^19^ Low-income areas and third-world countries are characterized by poverty, resulting in the consumption of cheaper food products.^20^ The Moli-sani study (n = 13 262) included a cohort from the Molise region of Italy.^17^ The main goal was to evaluate the risk factors for chronic degenerative disease, particularly cardiovascular and cerebrovascular diseases as well as cancer and neurodegenerative disorders. The study highlighted the impact of socio-demographic factors on adherence to the MeD by showing that participants in lower-income categories had poor adherence to the use of olive oil and vegetables and were more likely to adhere to a Western-type diet. On the other hand, participants with a higher income demonstrated better adherence to the MeD in terms of increased consumption of ﬁsh, fruits, and legumes and lower consumption of animal fats, processed meat, and white meat.^17^

In South Africa (SA), a developing country, most of the population has adopted a Western eating pattern.^20^ In the Free State province (the current study’s region), high intake of sugar, brick margarine, sunflower oil, and salt; and low intake of fruit and vegetables have been reported.^21^ In view of the health benefits of the MeD throughout the life course, the study aimed to determine adherence to the MeD and associated socio-economic factors amongst pregnant women in Bloemfontein, SA. We hypothesized that very few participants in our resource-limited setting would have good MeD adherence, and that socio-economic factors are associated with MeD adherence.

## Methodology

### Study design

A cross-sectional study was conducted.

### Sample selection

This sub-study formed part of an umbrella study, the Nutritional status of Expectant Mothers and their newborn Infants (NuEMI) study, that assessed the nutritional status of pregnant women between the ages of 18 and 44 years who visited the antenatal clinic at a Regional Hospital in central SA from May 2018 to April 2019.

### Ethical consideration

The study was approved by the Health Sciences Research Ethics Committee (UFS-HSD2018/0148/2905) at the University of the Free State and the Free State Department of Health. All participants signed written informed consent.

### Data collection

Trained fieldworkers completed questionnaires during structured interviews with participants. Socio-demographic information included age, the highest level of education, employment status, household income per month, and stability of household income. Dietary information was collected using a quantified food frequency questionnaire (QFFQ). The QFFQ has been validated earlier for the population in the Transition and Health during Urbanisation of South Africans (THUSA) study^22^ and the Women’s Health Study in the Free State^23^ and has shown very good reproducibility.^23-25^ Dietary intake was determined for the previous 28 days, and nutrient intake values were divided by 28 to calculate daily intake.^26^ Daily or weekly (28 days intake divided by 4) intake of relevant food groups included in the MeDAS 14-item questionnaire^14^ were extracted from the QFFQ. The MeDAS^14^ was adapted for the current study on pregnant women to a 13-item questionnaire; the question on alcohol (wine) was omitted.

Adherence to the MeDAS was assessed by applying a scoring system. If the food was consumed according to adherence amounts, 1 point was allocated, and if not, a point of 0 was allocated.^14^ A score was calculated by adding the points allocated to each of the 13 items on the list. As in previous studies in pregnant populations, the higher the score, the greater the adherence to the MED; classified as poor (⩽7 points), moderate (8-9 points), or good (⩾10 points).^4,6^

### Data analysis

Statistical analysis was performed by the Department of Biostatistics at the University of the Free State. Results were analyzed using SAS/STAT software, Version 9.4 of the SAS system for Windows, Copyright © 2010 SAS institute Inc. Descriptive statistics, namely frequencies and percentage for categorical data and medians and percentiles for numerical data were calculated. Associations between adherence to the Mediterranean diet and chosen factors in the existing dataset (socio-demographic information, including the level of education, employment status, and household income bracket) were calculated and described by means of Chi-square or Fisher’s exact test.

## Results

A total of 681 pregnant women participated with a median age of 31.8 years (IQR: 26.8-36.5 years) and a median gestational age of 32.0 weeks at the time of the interview. The median MeDAS adherence score was 5 points (IQR: 4-5), falling in the poor adherence to the Mediterranean diet category. Almost all participants (99.6%) had poor adherence (MeDAS score ⩽7) and only 0.4% (n = 3) had moderate adherence (MeDAS score 8-9), while no one had good adherence (MeDAS score 10-13) (Table 1).

**Table 1.** MeDAS score and socio-demographic information.

Total MeDAS^14^ score out of 13 (n = 681)	Number of participants (n)	Percentage (%)
1	1	0.2
2	11	1.6
3	56	8.2
4	218	32.1
5 (median score)	276	40.5
6	98	14.4
7	18	2.6
8	3	0.4
Socio-demographics		
Level of education (n = 681)	n	Percentage (%)
None	1	0.1
Primary school	47	6.9
Grade 8-10	181	26.6
Grade 11-12	373	54.8
Tertiary education	78	11.5
Don’t know	1	0.1
Household income per month (n = 679)		
None	2	0.3
R100 (7 USD)-R500 (33 USD)	29	4.3
R501 (33.3 USD)-R1000 (67 USD)	58	8.5
R1001 (67 USD)-R3000 (201 USD)	204	30.0
R3001 (201 USD)-R5000 (333 USD)	160	23.6
Over R5000 (>333 USD)	200	29.5
Don’t know	26	3.8
Employment status (n = 681)		
Full time employed	141	20.8
Part time employed	76	11.2
Unemployed	357	52.4
Self-employed	54	7.9
Housewife by choice	34	4.9
Other	19	2.8
Stability of income (past 6 months) (n = 664)		
Current income is more than the usual income received (in the past 6 months)	123	18.5
Current income is less than the usual income received (in the past 6 months)	156	23.6
Current income is the same as usual income received (in the past 6 months)	385	57.9

About half (54.8%, n = 373) of the group had an education up to secondary level (grade 11-12) (Table 1). More than 40% (43.2%, n = 293) received R3000 (201 United States Dollars [USD]) or less household income per month. The national minimum wage is R21.69 /hour^27^ (1.49 USD/hour), which calculates to approximately R3578 (246 USD) per month. This amount is sufficient for the survival of an individual and is above the poverty line of R1268 pm/pp^27^ (87 USD pm/pp). However, one should take into account that in this population, it is expected that this income will support multiple family members within the household, and therefore, this allocated amount may not be sufficient for food costs (Table 1).

Half (52.4%, n = 357) were unemployed and only one-fifth (20.7%, n = 141) were employed full time (Table 1). Almost a quarter (23.6%, n = 156) of the population had an unstable household income (current income is less than usual income received for the past 6 months).

Poor adherence scores were obtained due to the vast majority of participants not meeting the MeD guidelines with regard to using olive oil as the main culinary oil and not regularly eating sufficient amounts of vegetables, fruit, legumes, fish or shellfish, nuts, and sofrito (Table 2). However, a large percentage of participants succeeded in meeting the adherence requirements with regards to low consumption of red meat, saturated fat intake (butter, margarine, or cream), sweetened and carbonated beverages, commercial sweets or pastries, and higher consumption of chicken instead of veal, pork, hamburger, or sausage (Table 2).

**Table 2.** Scoring per question according to the MeDAS tool (n = 681).^14^

Variable	Number of participants (n)	Percentage (%)
Do you use olive oil as main culinary fat?		
Non-adherence: No	671	98.5
Adherence: Yes	10	1.5
How much olive oil do you consume in a given day (including oil used for frying, salads, out-of-house meals, etc.)?		
Non-adherence: <4 tbsps. (<60 ml)	638	93.7
Adherence: ⩾4 tbsps. (⩾60 ml)	43	6.3
How many vegetable servings do you consume per day?		
Non-adherence: <400 g	681	100.0
Adherence: ⩾400 g	0	0.0
How many fruit units (including natural fruit juices) do you consume per day		
Non-adherence: <360 g	678	99.6
Adherence: ⩾360 g	3	0.4
How many servings of red meat, hamburger, or meat products (ham, sausage, etc.) do you consume per day?		
Non-adherence: >150 g	1	0.2
Adherence: ⩽150 g	680	99.8
How many servings of butter, margarine, or cream do you consume per day?		
Non-adherence: >12 g	175	25.7
Adherence: ⩽12 g	506	74.3
How many sweet or carbonated beverages do you drink per day?		
Non-adherence: >330 ml (>1 serving)	28	4.1
Adherence: ⩽330 ml (⩽1 serving)	653	95.9
How many servings of legumes do you consume per week?		
Non-adherence: <3 servings (<450 g)	652	95.7
Adherence: ⩾3 servings (⩾450 g)	29	4.3
How many servings of fish or shellfish do you consume per week?		
Non-adherence: <3 servings (<300-450 g)	659	96.8
Adherence: ⩾3 servings (⩾300-450 g)	22	3.2
How many servings of nuts (including peanuts) do you consume per week?		
Non-adherence: <3 servings (<90 g)	637	93.5
Adherence: ⩾3 servings (⩾90 g)	44	6.5
How many times per week do you consume commercial sweets or pastries (not homemade), such as cakes, cookies, biscuits, or custard?		
Non-adherence: >3 servings (>150 g)	79	11.6
Adherence: ⩽3 servings (⩽150 g)	602	88.4
Do you preferentially consume chicken, turkey, or rabbit meat instead of veal, pork, hamburger, or sausage?		
Non-adherence: No	260	38.2
Adherence: Yes	421	61.8
How many times per week do you consume vegetables, pasta, rice, or other dishes seasoned with sofrito (sauce made with tomato and onion, leek, or garlic and simmered with olive oil)?		
Non-adherence: <60 ml (<2 servings)	514	75.5
Adherence: ⩾60 ml (⩾2 servings)	167	24.5

The amount of food consumed per week or day and the median intakes for the whole group are indicated in Table 3. The low vegetable intake of 17.0 g per day is especially concerning as the recommended intake is ⩾400 g/day^14^; in addition, 2 out of 5 did not consume vegetables at all (43.0%, n = 293).

**Table 3.** Amount of food consumed per week/day.^14^

Variables	Suggested intake	Minimum	Median	Maximum
Amount of olive oil (ml) per day (including oil used for frying, salads, out-of-house meals, etc.) (n = 652)	⩾60 ml	0.2	17.1	120.0
Amount of vegetables (g) consumed per day (n = 388)	⩾400 g	1.6	17.0	107.1
Amount of fruit (g) consumed per day (n = 656)	⩾360 g	0.9	32.9	571.4
Amount of red meat, hamburger, or meat products (ham, sausage, etc.) (g) consumed per day (n = 636)	⩽150 g	0.2	5.7	153.6
Amount of butter, margarine, or cream (g) consumed per day (n = 513)	⩽12 g	0.2	8.6	75.0
Amount of sweet and carbonated beverages (ml) per day (n = 387)	⩽330 ml	3.6	71.4	2000.0
Amount of legumes (g) consumed per week (n = 422)	⩾450 g	5.3	100.0	1800.0
Amount of fish or shellfish (g) consumed per week (n = 483)	⩾300-450 g	2.5	40.0	840.0
Amount of commercial sweets or pastries (not homemade), such as cakes, cookies, biscuits, or custard consumed per week (g) (n = 623)	⩽150 g	1.3	45.0	500.0
Amount of nuts (g) (including peanuts) consumed per week (n = 216)	⩾90 g	1.8	34.6	320.0
Amount of chicken (g) per day (n = 639)		16.0	276.0	4000.0
Amount sofrito (ml) per week (sauce made with tomato and onion, leek, or garlic and simmered with olive oil) (n = 246)	⩾60 ml	5.3	69.0	656.0

Compared to those with an income ⩽ R3000 (⩽201 USD), those with an income above R3000 (>201 USD) were significantly more likely to eat nuts (including peanuts) (⩾90 g per week) (2.0% vs 4.6%, *P* = .05), and adhere to sofrito intake (tomato and onion relish similar to sofrito a sauce made with tomato and onion, leek, or garlic and simmered with olive oil) (⩾60 ml per week) (9.2% vs 15.6%, *P* = .02) (Table 4).

**Table 4.** Frequency distribution of MeD adherence according to income and education.

Variable	Receiving ⩽ R3000 pm (⩽201 USD)		Receiving > R3000 pm (>201 USD)				*P*-value
	n	Percentage (%)	n	Percentage (%)			
How many servings of nuts (including peanuts) do you consume per week? (n = 653)							
Non-adherence: <3 servings (<90 g)	280	95.6	330	91.7			.05
Adherence: ⩾3 servings (⩾90 g)	13	4.4	30	8.3			
How many times per week do you consume vegetables, pasta, rice, or other dishes seasoned with sofrito (sauce made with tomato and onion, leek, or garlic and simmered with olive oil)? (n = 653)							
Non-adherence: <60 ml (<2 servings)	233	79.5	258	71.7			.02
Adherence: ⩾60 ml (⩾2 servings)	60	20.5	102	28.3			
	Primary education—grade 10		Grade 11 up to tertiary education				*P*-value
	n	Percentage (%)	n	Percentage (%)			
How much olive oil do you consume in a given day (including oil used for frying, salads, out-of-house meals, etc.)? (n = 681)							
Non-adherence: <4 tbsps (<60 ml)	220	96.1	418	92.5			.01
Adherence: ⩾4 tbsps. (⩾60 ml)	9	3.9	34	7.5			
How many times per week do you consume vegetables, pasta, rice, or other dishes seasoned with sofrito (sauce made with tomato and onion, leek, or garlic and simmered with olive oil)? (n = 681)							
Non-adherence: <60 ml (<2 servings)	184	80.4	330	73.0			.02
Adherence: ⩾60 ml (⩾2 servings)	45	19.6	122	27.0			
	Current income is more than the usual income received (in the past 6 months)		Current income is less than the usual income received (in the past 6 months)		Current income is the same as usual income received (in the past 6 months)		*P*-value
	(n)	Percentage (%)	(n)	Percentage (%)	(n)	Percentage (%)	
How many sweet or carbonated beverages do you drink per day? (n = 664)							
Non-adherence: >330 ml (>1 serving)	3	2.4	2	1.3	22	5.7	.04
Adherence: ⩽330 ml (⩽1 serving)	120	97.6	154	98.7	363	94.3	

Compared to those who only had a primary education level up to grade 10 (n = 229), those who had a secondary education level or more (grade 11 and higher) (n = 452) were significantly more likely to consume enough olive oil per day (⩾60 ml/day) (1.3% vs 5.0%, *P* = .01), although in both groups only a few consumed enough olive oil, and sofrito (⩾60 ml per week) (6.6% vs 18.0%, *P* = .02) (Table 4).

Compared to those who did not have a stable income (receiving less than usual in the past 6 months), those that had a stable income were significantly more likely to consume >330 ml/day sweet or carbonated beverages (0.3% vs 3.3%, *P* = .04), albeit only 27 participants in total consumed more than 330 ml/day (Table 4).

## Discussion

Although several studies have assessed adherence of certain groups^4,6,28^ to the MeD, none seem to have been undertaken in SA.

Adherence to the MeD was poor amongst the sample of pregnant women included in the current study (with 99.6% scoring less than 5 out of 13).^14^ Findings of other studies have reported low to moderate adherence to the MeD in pregnant women. A large United States and Greek cohort of pregnant women (N = 1566) reported mean scores of 2.7 and 3.8 out of 9, respectively, using the MDS.^6^ The education level in these 2 cohorts was generally high, with 71% having a college degree, although it did not increase adherence.^6^ Using the MEDSS, a study in Croatia^28^ among 266 pregnant women found good adherence in 27.8% of participants, which is higher than in the current study. Havaš Auguštin et al^28^ further concluded that good adherence was associated with higher wealth. In the current study, those having a higher income were significantly more likely to adhere to nut and sofrito intake.^28^

According to Vilarnau et al^29^ (2019) there has been a global decline in the adherence to the MeD from the 1960s to the early 21st century. The leading cause of this is due to eating habits becoming more Western. The latest research furthermore suggests that the last decade’s economic crisis could be an additional contributing factor.^29^ During the planning stages of the study, we expected that income and level of education would significantly impact the overall adherence to the MeD. However, this was not the case. Although level of education was not associated with improved adherence to overall adherence to the MeD in the current study (possibly because almost all participants had poor adherence), this does not mean that education does not influence adherence. In fact, it is possible to be educated and still have a nutritional knowledge deficit, as in the case of South Africa, where only 1 in 5 participants (22.6%) achieved a high score in general nutrition knowledge score,^20^ which in turn, could influence lifestyle choices and decisions regarding food intake. Conversely, nutrition knowledge alone does not necessarily lead to improved outcomes; practices are also dependent on food availability, cultural practices, and the economic environment, amongst others.^30^

The associations between intake of nuts, sofrito, and olive oil with income and education confirm that adherence is influenced by wider socio-demographic determinants. In the South African setting Ronquest-Ross^31^ has identified that food intake is affected by factors such as geography, season, education, disposable income, urbanization, globalization, marketing, religion, culture, ethnicity, social networks, consumer preference, and time. According to Micklesfield et al,^32^ South Africans’ urbanization into informal communities is becoming more prevalent, which means that the most common place to buy food is from informal vendors. Furthermore, purchasing foods from vendors and local tuck shops has led to a decline in grocery-style shopping.^32^ This has resulted in new food purchasing habits, including a higher intake of foods with less nutritional value.^32^ In light of the above, dietary recommendations need to take the general living environment in the South African setting into account to ensure sustainable improvements in dietary intake.

We indicated poor adherence to the MeD in the current study, specifically related to the components olive oil not used as the main culinary oil, inadequate intake of fruit, vegetables, legumes, fish or shellfish, nuts, and sofrito. The MeD seems foreign to the South African culture, which currently favors a more Western eating pattern.^21^ Dietary intake data from the Assuring Health for All in the Free State (AHA-FS) study,^21^ Women’s Health Study,^33^ and the THUSA study^25^ show that foods that are commonly eaten in the region include few foods preferred on a MeD. Foods eaten most frequently include, amongst others, sunflower oil,^21,25^ white bread,^21,25^ maize meal porridge (pap),^21,25,33^ table sugar,^21,25,33^ sweetened cold drinks,^21,25,33^ crisps,^33^ and biscuits.^33^ Therefore, healthy alternatives to the items included in the original MeDAS tool^14^ that are culturally acceptable such as pilchards, tuna, fruit, and nuts (eg, peanuts^21^), canola oil (instead of sunflower oil), could provide beneficial characteristics similar to those of the MeD such as fiber, antioxidants, monounsaturated fatty acids (MUFA), and omega-3 fatty acids.^9^ Since the intake of vegetables in the current study was low, it would be of great benefit to encourage vegetable gardening as this could contribute to continuous and sustainable access to vegetables as well as improving attitudes toward, willingness to taste, and self-efficacy to prepare/cook fruit and vegetables.^34^ Prioritizing dietary education^35^ is also a very important factor to consider as some foods which are regularly consumed in the SA setting are processed and expensive.^20^ The low intake of olive oil could be addressed by substituting olive oil with a broader range of cheaper mono-unsaturated fatty acids (MUFA’s) that also have a beneficial fatty acid composition. Cheaper sources of MUFAs such as canola oil (70 ml canola oil = 43.8 g MUFA [similar to MUFA content in 60 ml olive oil]),^36^ peanut butter, peanuts, and pumpkin seeds^37^ are also good sources of MUFA’s. Consequently, the researchers propose the development of a contextualized MeDAS tool to be used in SA.

Although the exact foods listed in the original MeDAS^14^ may not be included, the adapted guidelines could have similar health benefits, while also being culturally acceptable. Dietary intake data obtained from food frequency questionnaires in the AHA-FS study,^21^ SA Women’s Health Study,^33^ and the THUSA study^25^ can be used to include foods that are frequently eaten in the region in an adapted MeDAS tool to contextualize it for SA. Before such a tool could be widely applied, it would have to be validated in the SA population.

## Limitations

The MeDAS^14^ used in the current study, was developed for European populations and has not been validated for use in South Africa.

The group of participants with moderate to poor adherence was very small, making it difficult to determine associations. Future studies should consider including participants from other regions of South Africa, as this would provide a more representative sample.

## Conclusion and Recommendations

We showed support for the hypothesis that very few pregnant women in our resource-limited setting had good MeD adherence. The current study provided an intriguing insight into adherence to the MeD diet in the African culture. To date, similar studies have only been conducted in developed countries, which is a strength of the current study that provides new and valuable insight into adherence to the MeD in a developing country in the African region.

Income and education were associated with the intake of specific components of the diet, confirming that socio-demographic factors impact on adherence. In light of the benefits associated with the MeD, recommendations to increase adherence to the principles of the MeD by adapting them to the South African context are justified.

The Social Cognitive Theory (SCT)^38^ suggests that for an individual to perform a particular behavior, they should be aware of the behavior and how to achieve it.^18^ Considering the theory, an improved understanding of the MeD is required to improve adherence, including the impact of social and cultural determinants of health.^18^
